# Biodiversity and Ecosystem Multi-Functionality: Observed Relationships in Smallholder Fallows in Western Kenya

**DOI:** 10.1371/journal.pone.0050152

**Published:** 2012-11-28

**Authors:** Jason Sircely, Shahid Naeem

**Affiliations:** Department of Ecology, Evolution, and Environmental Biology, Columbia University, New York, New York, United States of America; University of Zurich, Switzerland

## Abstract

Recent studies indicate that species richness can enhance the ability of plant assemblages to support multiple ecosystem functions. To understand how and when ecosystem services depend on biodiversity, it is valuable to expand beyond experimental grasslands. We examined whether plant diversity improves the capacity of agroecosystems to sustain multiple ecosystem services—production of wood and forage, and two elements of soil formation—in two types of smallholder fallows in western Kenya. In 18 grazed and 21 improved fallows, we estimated biomass and quantified soil organic carbon, soil base cations, sand content, and soil infiltration capacity. For four ecosystem functions (wood biomass, forage biomass, soil base cations, steady infiltration rates) linked to the focal ecosystem services, we quantified ecosystem service multi-functionality as (1) the proportion of functions above half-maximum, and (2) mean percentage excess above mean function values, and assessed whether plant diversity or environmental favorability better predicted multi-functionality. In grazed fallows, positive effects of plant diversity best explained the proportion above half-maximum and mean percentage excess, the former also declining with grazing intensity. In improved fallows, the proportion above half-maximum was not associated with soil carbon or plant diversity, while soil carbon predicted mean percentage excess better than diversity. Grazed fallows yielded stronger evidence for diversity effects on multi-functionality, while environmental conditions appeared more influential in improved fallows. The contrast in diversity-multi-functionality relationships among fallow types appears related to differences in management and associated factors including disturbance and species composition. Complementary effects of species with contrasting functional traits on different functions and multi-functional species may have contributed to diversity effects in grazed fallows. Biodiversity and environmental favorability may enhance the capacity of smallholder fallows to simultaneously provide multiple ecosystem services, yet their effects are likely to vary with fallow management.

## Introduction

Biodiversity in its broad sense is responsible for the ecosystem services relied on by human societies [1], [2]. However, the linkages among biodiversity and many ecosystem services remain poorly understood. Biodiversity conservation strategies tend to be based on intrinsic and cultural values [3], rather than these values in combination with use values [4], [5]. Either under- or over-estimating the utility of biodiversity to stakeholders may threaten conservation efforts. Empirical information on biodiversity relationships with ecosystem services may ultimately assist conservation of biodiversity and alleviation of rural poverty in developing countries. If the cumulative values attributable to biodiversity are more difficult to replace than managers expect, management decisions that excessively discount returns to biodiversity may impede ecosystem service delivery.

In recent decades a growing body of evidence from manipulative experiments has demonstrated that diversity in ecological communities can significantly influence ecosystem functioning [6], [7], [8], [9]. Plant diversity manipulations, primarily in grasslands, have shown that diversity effects on ecosystem functions such as biomass production and nutrient retention are typically positive and saturating at high species richness [6], [8], [9], [10].

Biodiversity effects on ecosystem function are generally attributed to complementarity effects and sampling (or selection) effects. Complementarity effects reflect the net outcome of all biological processes affecting the functioning of an assemblage [8], for instance through niche partitioning among species reducing competition. Sampling effects arise from the higher probability of species efficacious for a particular function being over-represented at higher species richness. In grasslands, complementarity effects may be primarily responsible for diversity effects on productivity [8], [10].

Biodiversity-ecosystem function research is currently expanding to address multiple ecosystem functions and a broader range of ecosystems. From a more theoretical perspective, the focus on diversity effects on individual ecosystem functions does not embrace the likelihood that species affect different ecosystem functions differently [11]. From a more ‘real-world’ perspective, the emphasis on mesocosms mimicking natural grasslands limits applicability to ecosystems in general. Ultimately, if biodiversity is to be linked with ecosystem services in natural and managed systems, biodiversity-ecosystem function research will account for differences between experimental grasslands and, for example, agroecosystems and forests that provide ecosystem services essential to society.

While relatively few studies have addressed plant diversity effects on terrestrial ecosystem function outside of grasslands, a handful of studies have investigated whether plant diversity enhances the capacity to provide multiple functions simultaneously, i.e., ecosystem multi-functionality.

### Biodiversity and ecosystem multi-functionality

Evidence for a greater ability of diverse plant assemblages to support multiple ecosystem functions has emerged from grassland diversity manipulation studies. These studies tested distinct yet related hypotheses: (1) considering a greater number of functions increases the number of species important for functioning [11]; (2) species richness increases the probability that several functions are sustained at half-maximum [12]; (3) considering a greater number of functions increases the minimum richness required for half of assemblages to sustain all functions above a certain threshold; and also, the proportion of assemblages achieving thresholds increases with richness [13]. The functions considered ranged from plant biomass production and indicators of N uptake to primary consumer production, soil functions, and invasion resistance. All three studies concluded that species richness enhanced the capacity to sustain multiple functions. Meanwhile, a global empirical study in drylands recently found positive effects of species richness on indices of multi-functionality based on biogeochemical indicators, in spite of vast abiotic variation [14].

Complementarity among plant species, both within as well as among ecosystem functions, may significantly underlie plant diversity effects on ecosystem multi-functionality. That is, plant diversity may improve multi-functionality in part through complementarity effects specific to individual ecosystem functions. Meanwhile, different species providing complementary benefits to different ecosystem functions should further enhance multi-functionality. ‘Multi-functional’ species able to support more than one function may also promote multi-functionality. Some agroforestry trees in our study area are multi-functional: Sesbania sesban (L.) Merr., for instance, is productive, can fix over 70% of its nitrogen content from the atmosphere, and can prevent leaching of soil NO_3_^−^ [15]. Multi-functional species may contribute to diversity effects on multi-functionality by uniquely combining functional traits—disproportionately increasing functional diversity—or by their representation increasing probabilistically with species richness.

### Biodiversity and agroecosystem multi-functionality

In agroecosystems, plant diversity might influence multiple ecosystem functions more strongly than in experiments. Management practices such as harvesting, grazing, and land use rotations cause significant disturbance. Stronger effects of diversity on ecosystem function in the presence of disturbance [16], [17] could similarly enhance the multi-functionality of managed systems.

The potential of biodiversity to buffer ecosystem functioning against environmental change is known as the insurance hypothesis [18], [19], and enhanced resilience is a key rationale for the retention of biodiversity in agroecosystems [20], [21]. Disturbance may increase the significance of the diversity in functional traits mediating responses to the perturbation. Species that can tolerate disturbance or re-colonize afterward will subsequently contribute to ecosystem function, and the pre- and post-disturbance assemblages will likely function differently.

When a biotic community experiences a perturbation, the importance of functional trait diversity depends on whether the traits engendering vulnerability (response traits) also determine species effects on ecosystem function (effect traits). When species with strong effects are more vulnerable, ecosystem function declines more severely than with random species loss [22]. For instance, larger bee species effective as pollinators can be more extinction-prone, causing a disproportionate decline in pollination with species loss [23]; aboveground C stocks should decline rapidly when timber extraction favors trees of high wood density [24]. Hence, biodiversity-ecosystem function research is attentive not only to functional traits determining ecosystem effects, but also traits mediating responses to environmental change [25], [26].

Plant responses to aboveground biomass removal are mediated by traits associated with two main strategies: tolerance and avoidance. The first suite of traits enables plants to withstand disturbance, such as resprouting ability [27], [28], vegetative reproduction, belowground storage, low meristems, seed dormancy, and perenniality [29]. The second suite bestows high fecundity and growth rates which facilitate re-establishment. Annual plants typify avoidance, with traits such as small seeds that enhance dispersal [29], and rapid growth traits including high specific leaf area (SLA; area per unit mass) and leaf N [30].

At global scales, large herbivore grazing tends to favor annuals (indicating avoidance) and short, prostrate, or stoloniferous plants (indicating tolerance) [31]. Forbs typically decline, while species less palatable—e.g., due to low SLA and leaf N [32]—often increase. In contrast, the palatability of grasses in humid and sub-humid regions can increase during overcompensation, a dramatic rise in growth rates following grazing [33], [34]. Thus, the responses of palatability to grazing vary widely, and may significantly alter rates of ecosystem processes, including by shifting litter chemistry and productivity.

In addition to disturbance, tropical smallholder agroecosystems have further characteristics that may increase the likelihood of plant diversity influencing ecosystem multi-functionality. Smallholder farms tend to be more multi-functional [35] than commercial ones, with species ranging from annual and perennial crops to livestock forages, to trees grown for wood products and tree-crops. Smallholders in the tropics often plant multi-functional species yielding multiple benefits, including multi-purpose trees that improve soil quality while producing valuable goods [15], [36]. Others are perennial crops: cassava (*Manihot esculenta* Crantz), for instance, is a major food crop globally [37] and can improve soil fertility compared to annual crops [38]. Multi-functional species may be exotic to a system, or native species planted or encouraged to regenerate (e.g., *Sesbania* in our study site).

The goal of the current study is to assess whether plant diversity and environmental conditions influence the capacity of tropical smallholder fallows to provide multiple ecosystem services. A set of four ecosystem functions were selected as indicators for the ecosystem services of wood production, livestock forage production, and soil formation, the latter of which affects post-fallow crop production. Ecosystem service multi-functionality was quantified as (1) the proportion of ecosystem functions above half-maximum (henceforth, “proportion above half-maximum”), and (2) mean percentage excess above mean ecosystem function values (“mean percentage excess”). Two specific hypotheses were tested: first, plant diversity increases the ecosystem multi-functionality of fallows; and second, multi-functionality increases with environmental favorability, in terms of lower grazing intensity (in grazed fallows only) and higher soil fertility, since favorable environmental conditions may benefit each ecosystem function. Here we present the first assessment of plant diversity influences on multiple ecosystem functions in the humid tropics, and the first test of biodiversity influences on multiple ecosystem functions directly linked with ecosystem services relevant to smallholder farmer livelihoods.

## Results

**Figure 1:**
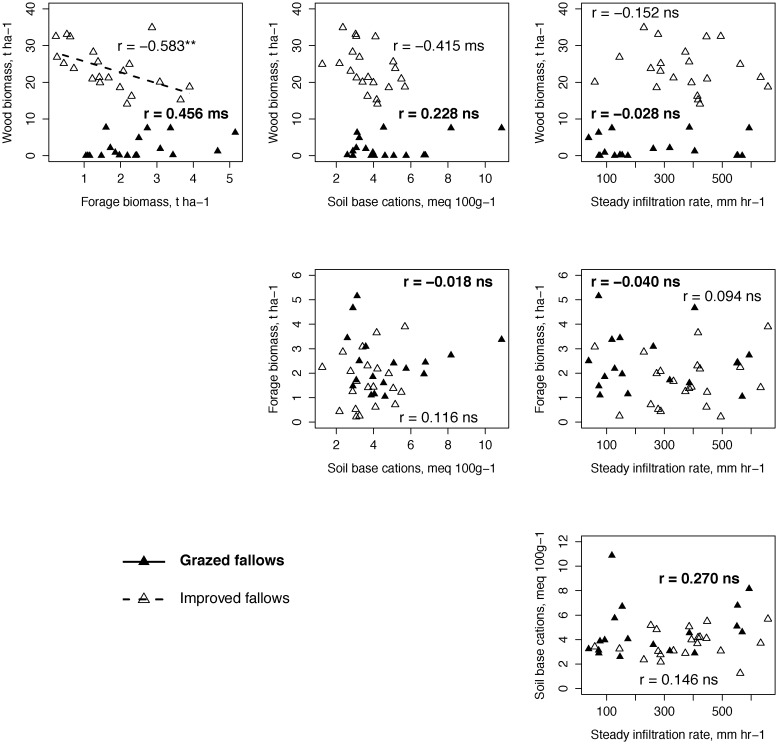
Bi-variate correlations among fallow ecosystem functions; * *P*<0.05, ** *P*<0.01, ms *P*<0.1, ns *P*>0.1 for Pearson's *r*.

Correlations among the selected set of four ecosystem functions were weak in both grazed and improved fallows, with the exception of the negative correlation between wood biomass and forage biomass in improved fallows. Wood and forage biomass were positively and marginally significantly correlated (Figure 1) in grazed fallows. In improved fallows, wood biomass exhibited a marginally significant negative correlation with soil base cations. Correlations were insignificant (at α = 0.05) for all other pairs of ecosystem functions (Figure 1).

Ecosystem multi-functionality, in terms of the proportion of ecosystem functions above half-maximum (“proportion above half-maximum”) and mean percentage excess above mean function values (“mean percentage excess”), varied more widely in grazed than in improved fallows (Table 1). The proportion of functions above 25, 50, and 75% of function maxima was higher in improved than in grazed fallows, most consistently at the 25 and 50% levels (Figure S1); mean percentage excess cannot, by definition, differ on average among fallow types. Only one improved fallow sustained more than two functions above 75% of maxima.

**Table 1:** Fallow ecosystem functions serving as indicators of ecosystem services (ES), and indicators of ecosystem multi-functionality among the four ecosystem functions.

Indicator	Indicator of:	Units (Transformation)	Fallow type	n	Mean	SD	SEM	Max^a^
Wood biomass	Wood production ES	t ha^−1^	Grazed	18	3.0	3.3	0.8	8.0
		(natural log)	Improved	21	24.2	5.9	1.3	31.1
Forage biomass	Forage production ES	t ha^−1^	Grazed	18	2.4	1.2	0.3	4.4
		(square root)	Improved	21	1.7	1.1	0.2	3.5
Soil base cations	Soil formation ES	meq 100 g^−1^	Grazed	18	4.8	2.2	0.5	8.8
		(arcsine square root)	Improved	21	3.7	1.2	0.3	5.6
Steady infiltration rate	Soil formation ES	mm hr^−1^	Grazed	18	261.8	198.1	46.7	571.7
		(square root)	Improved	21	371.0	148.1	32.3	617.3
Proportion of ecosystem functions above half-maximum	Ecosystem multi-functionality	%	Grazed	18	31.9	25.5	6.0	na
		(arcsine square root)	Improved	21	56.0	17.5	3.8	na
Mean percentage excess above mean ecosystem function values	Ecosystem multi-functionality	Mean %	Grazed	18	0.0	33.0	7.8	na
		(none)	Improved	21	0.0	8.6	1.9	na

a The maximum of each ecosystem function is the mean of the 3 highest values in a given fallow type.

Grazed fallows had substantially higher plant diversity than improved fallows, both in terms of the species richness (ANOVA, d.f. = 1,37; *F* = 12.2; *P*<0.01) and functional diversity (ANOVA, d.f. = 1,37; *F* = 21.3; *P*<0.0001) of fallow plants (Table S1).

### Ecosystem multi-functionality: Grazed fallows

**Figure 2:**
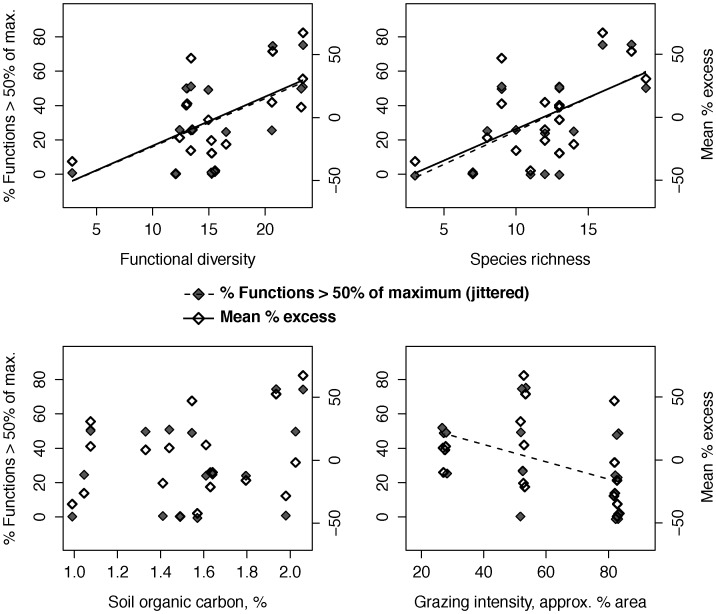
Grazed fallows: Relationships of plant diversity and environmental variables with ecosystem multi-functionality indicators.

In grazed fallows, the proportion of functions above half-maximum and mean percentage excess exhibited similar relationships with plant diversity and environmental variables (Figure 2). Both the proportion above half-maximum (Table 2) and mean percentage excess (Table 3) were best explained by positive effects of fallow plant diversity, with functional diversity and species richness explaining similar proportions of the variance (functional diversity and species richness were highly correlated; d.f. = 1,16; *r* = 0.854; 95% CI [0.64, 0.94]). While the proportion above half-maximum declined with grazing intensity, mean percentage excess was not associated with grazing intensity, and neither indicator correlated with soil carbon (Tables 2, 3; Figure 2).

**Table 2:** Grazed fallows: Plant diversity and environmental correlates of the proportion of ecosystem functions above half-maximum.

Model specification	d.f.^a^	Intercept	Predictor	Est.	*t*	*P*	*R ^2^* b
Functional diversity	1,16	−0.10	Functional diversity	0.04	2.62	0.019	0.256
Species richness	1,16	−0.11	Species richness	0.05	2.71	0.016	0.271
Soil carbon	1,16	−0.07	Soil carbon	4.77	0.74	0.467	−0.027
Grazing intensity	1,16	1.18	Grazing intensity	−0.73	−2.15	0.047	0.176
Functional diversity+Soil carbon	2,15	−0.12	Functional diversity	0.04	2.39	0.030	0.207
			Soil carbon	0.15	0.03	0.980	
Species richness+Soil carbon	2,15	−0.02	Species richness	0.06	2.48	0.025	0.224
			Soil carbon	−0.81	−0.13	0.895	
Functional diversity+Grazing intensity	2,15	0.46	Functional diversity	0.03	1.91	0.075	0.293
			Grazing intensity	−0.46	−1.35	0.196	
Species richness+Grazing	2,15	0.43	Species richness	0.04	1.97	0.068	0.301
			Grazing intensity	−0.45	−1.30	0.214	

a d.f. = model, error degrees of freedom.

b *R^2^* = total model *R^2^* adjusted for number of parameters.

**Table 3:** Grazed fallows: Plant diversity and environmental correlates of mean percentage excess above mean ecosystem function values.

Model specification	d.f.^a^	Intercept	Predictor	Est.	*t*	*P*	*R^2^* b
Functional diversity	1,16	−61.08	Functional diversity	3.89	3.03	0.008	0.324
Species richness	1,16	−58.88	Species richness	5.00	2.88	0.011	0.301
Soil carbon	1,16	−71.40	Soil carbon	578.14	1.03	0.320	0.003
Grazing intensity	1,16	48.03	Grazing intensity	−53.42	−1.70	0.109	0.100
Functional diversity+Soil carbon	2,15	−76.13	Functional diversity	3.77	2.69	0.017	0.283
			Soil carbon	137.56	0.27	0.789	
Species richness+Soil carbon	2,15	−67.61	Species richness	4.89	2.53	0.023	0.255
			SOC	81.35	0.16	0.879	
Functional diversity+Grazing intensity	2,15	−32.24	Functional diversity	3.43	2.42	0.029	0.309
			Grazing intensity	−24.08	−0.80	0.436	
Species richness+Grazing	2,15	−29.62	Species richness	4.37	2.26	0.039	0.283
			Grazing intensity	−24.24	−0.78	0.445	

a d.f. = model, error degrees of freedom.

b *R^2^* = total model *R^2^* adjusted for number of parameters.

In linear models combining grazing intensity with plant diversity variables, functional diversity and species richness had marginally significant effects on the proportion above half-maximum, and remained significant predictors of mean percentage excess, while grazing intensity was not a significant predictor of either multi-functionality indicator (Tables 2, 3). For mean percentage excess, the inclusion of grazing intensity reduced the proportion of variance explained, compared to models with functional diversity or species richness as the sole predictor, despite the additional parameter. In models combining soil carbon with plant diversity variables, functional diversity and species richness were again significant predictors of both indicators, and soil carbon was again insignificant (Tables 2, 3). Grazing intensity was not correlated with functional diversity or species richness (d.f. = 1,16; respectively, *r* = −0.403; 95% CI [−0.73, 0.08] and *r* = −0.418; 95% CI [−0.74, 0.06]), nor was soil carbon (d.f. = 1,16; respectively, *r* = 0.324; 95% CI [−0.17, 0.69] and *r* = 0.374; 95% CI [−0.11, 0.72]).

**Figure 3:**
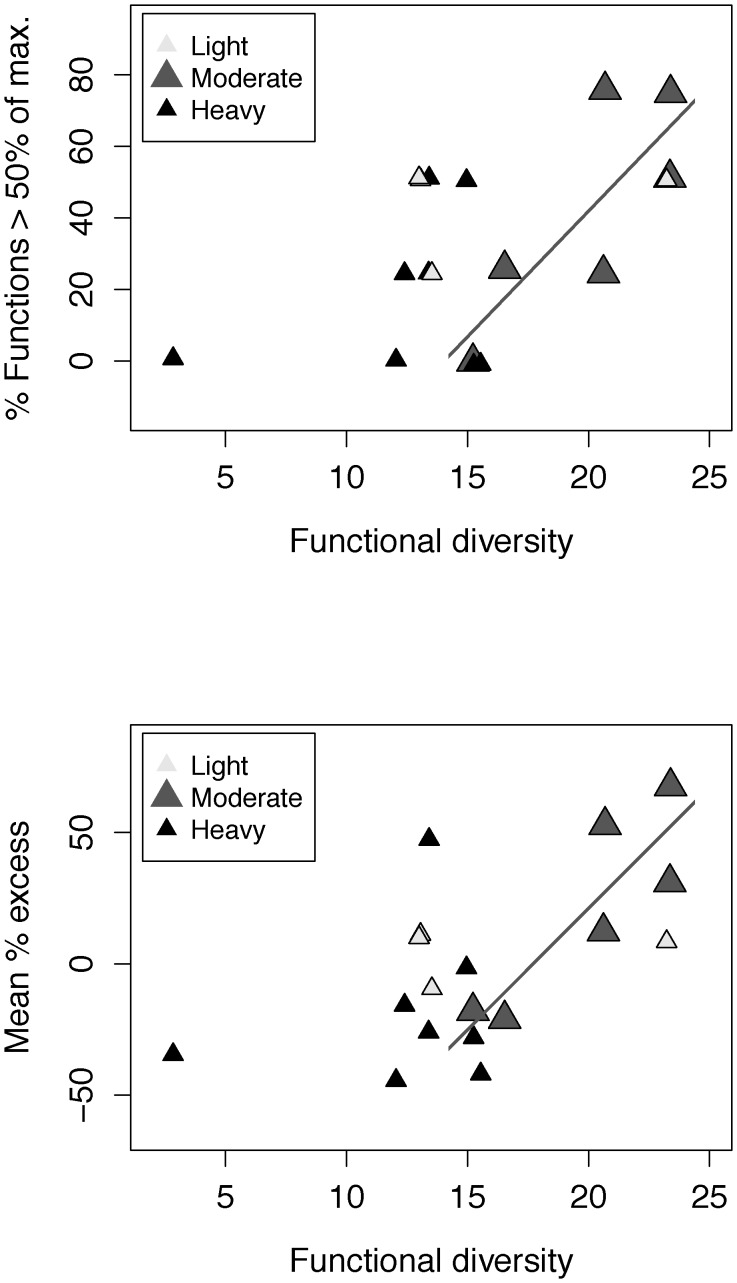
Grazed fallows: Plant functional diversity relationships with ecosystem multi-functionality indicators within grazing intensity groups.

Functional diversity was most evenly distributed at moderate grazing intensity (and was slightly higher), while the sample was small at low grazing intensity (n = 4), and diversity varied little at high grazing intensity. As such, the moderately grazed group (i.e., ∼50% of area with evidence of grazing) provided the most meaningful test of diversity effects on multi-functionality within a single grazing group. In moderately grazed fallows, the proportion above half-maximum (d.f. = 1,4; *R* ^2^ = 0.582; *P*<0.05) and mean percentage excess (d.f. = 1,4; *R* ^2^ = 0.688; *P*<0.05) again increased with functional diversity (Figure 3).

### Ecosystem multi-functionality: Improved fallows

**Figure 4:**
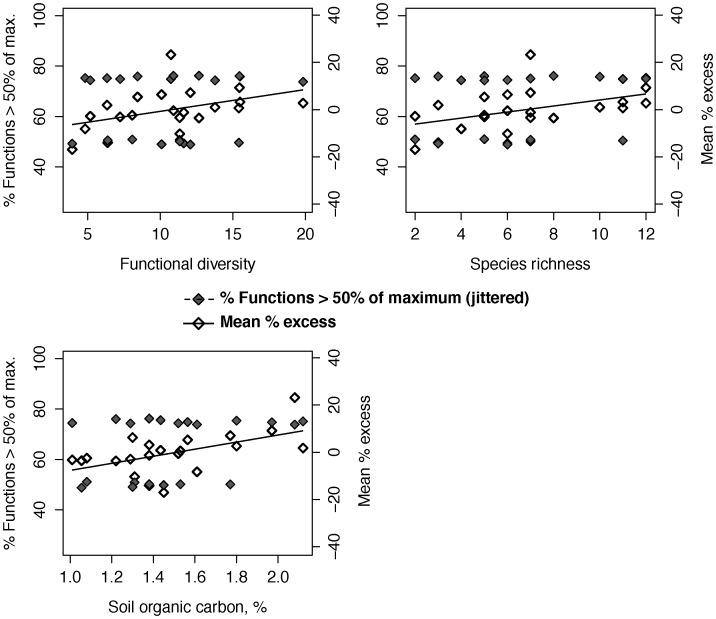
Improved fallows: Relationships of plant diversity and environmental variables with ecosystem multi-functionality indicators.

Improved fallows supported between two and three functions above 50% of function maxima, and between three and four functions above 25% of function maxima (Figure S1). Mean percentage excess did not diverge more than 25% above or below 0 (Figure 4).

In improved fallows, ecosystem multi-functionality exhibited considerably different relationships with plant diversity and environmental conditions depending on the multi-functionality indicator considered. The proportion above half-maximum was unassociated with fallow plant diversity or soil carbon (Table 4, Figure 4). Mean percentage excess increased with functional diversity, species richness, and soil carbon (Table 5, Figure 4), with soil carbon explaining the most variance. In models combining soil carbon with diversity variables, soil carbon was again the best predictor of mean percentage excess, while diversity variables were marginally significant (Table 5).

**Table 4:** Improved fallows: Plant diversity and environmental correlates of the proportion of ecosystem functions above half-maximum.

Model specification	d.f.^a^	Intercept	Predictor	Est.	*t*	*P*	*R^2^* b
Functional diversity	1,19	0.90	Functional diversity	0.003	0.47	0.642	−0.040
Species richness	1,19	0.89	Species richness	0.007	0.78	0.446	−0.020
Soil carbon	1,19	0.50	Soil carbon	3.612	1.62	0.121	0.076
Functional diversity+Soil carbon	2,18	0.49	Functional diversity	0.001	0.17	0.865	0.026
			Soil carbon	3.531	1.51	0.147	
Species richness+Soil carbon	2,18	0.49	Species richness	0.005	0.48	0.634	0.037
			Soil carbon	3.380	1.46	0.162	

a d.f. = model, error degrees of freedom.

b *R^2^* = total model *R^2^* adjusted for number of parameters.

**Table 5:** Improved fallows: Plant diversity and environmental correlates of mean percentage excess above mean ecosystem function values.

Model specification	d.f.^a^	Intercept	Predictor	Est.	*t*	*P*	*R^2^* b
Functional diversity	1,19	−9.73	Functional diversity	0.92	2.16	0.044	0.155
Species richness	1,19	−8.45	Species richness	1.28	2.29	0.034	0.175
Soil carbon	1,19	−43.36	Soil carbon	356.38	2.75	0.013	0.247
Functional diversity+Soil carbon	2,18	−45.27	Functional diversity	0.73	1.88	0.076	0.336
			Soil carbon	308.95	2.48	0.023	
Species richness+Soil carbon	2,18	−43.87	Species richness	1.02	2.01	0.059	0.351
			Soil carbon	305.23	2.48	0.023	

a d.f. = model, error degrees of freedom.

b *R^2^* = total model *R^2^* adjusted for number of parameters.

### Jointness among ecosystem functions

Patterns of ecosystem multi-functionality can be decomposed by assessing pair-wise jointness among ecosystem functions (see Supplement S1). Here, a pair of functions were considered to exhibit jointness when the observed values of both functions were above 50% of maximum (for the proportion above half-maximum), or above the mean (for mean percentage excess). Jointness was interpreted as reflecting the observed ability of a fallow to simultaneously sustain the pair of functions at or above moderate levels of each.

**Figure 5:**
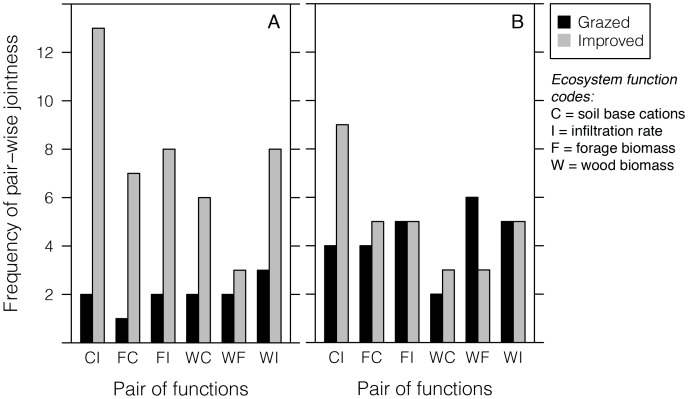
Incidence of jointness for pairs of ecosystem functions A) above 50% of function maxima, and B) above mean function values.

In improved fallows, the negative correlation between wood and forage biomass (Figure 1) was reflected in their low incidence of jointness, while in grazed fallows wood-forage jointness was not particularly low (Figure 5), in agreement with their weak positive correlation (Figure 1). Similarly, low jointness with respect to mean values for wood biomass and soil base cations in improved fallows may be attributed to a marginally significant negative correlation. Otherwise, there was no evidence that linear co-variance among ecosystem functions contributed to jointness, or by extension multi-functionality, in either fallow type. Jointness at 50% of maximum was at times attributable to the distributional characteristics of individual functions, e.g., for soil base cations and steady infiltration rates.

Soil base cations and infiltration rates jointly exceeded 50% of maxima or mean values in only a few grazed fallows. The probability distributions of base cations and infiltration were skewed left-ward and the incidence of jointness was lower than in improved fallows (Supplement S1). Grazing intensity did not correlate with soil carbon (d.f. = 1,16; *r* = 0.143; 95% CI [−0.35, 0.57]), soil base cations (d.f. = 1,16; *r* = 0.037; 95% CI [−0.44, 0.50]), or infiltration (d.f. = 1,16; *r* = −0.169; 95% CI [−0.59, 0.32]) in grazed fallows, although infiltration rates were lower in grazed than in improved fallows (ANOVA, d.f. = 1,37; *F* = 5.15; *P*<0.05). Soil carbon was correlated with base cations (d.f. = 1,16; *r* = 0.751; 95% CI [0.44, 0.90]) in grazed fallows, but not with infiltration (d.f. = 1,16; *r* = 0.301; 95% CI [−0.19, 0.67]).

In improved fallows, approximately half the sample exhibited jointness for soil base cations and infiltration rates (Figure 5), despite no correlation among them (Figure 1), the highest incidence of jointness observed. Base cations and infiltration had right-skewed distributions (Supplement S1) in improved fallows. Although soil carbon influences both, correlations of soil carbon with base cations and infiltration could not explain their jointness. That is, although infiltration was correlated with soil carbon (d.f. = 1,19; *r* = 0.446; 95% CI [0.02, 0.74]) in improved fallows, base cations were not (d.f. = 1,19; *r* = 0.240; 95% CI [−0.21, 0.61]).

### Grazed fallows: Functional traits and individual ecosystem functions

In grazed fallows, wood biomass increased with the abundance-weighted mean for green tissue lignin content of trees and shrubs (d.f. = 1,16; *r* = 0.562; 95% CI [0.05, 0.84]), and was not significantly associated with their contents of N or polyphenols. Forage biomass in grazed fallows increased with tissue N content of forage species (d.f. = 1,16; *r* = 0.584; 95% CI [0.16, 0.83]). Increased soil carbon and infiltration rates with green tissue content of recalcitrant C compounds, i.e., lignins+polyphenols, were documented in the study fallows (Sircely, *unpublished data*). Although soil carbon was closely correlated with soil base cations (d.f. = 1,16; *r* = 0.751; 95% CI [0.44, 0.90]), base cations and content of lignins+polyphenols were not significantly correlated in grazed fallows (d.f. = 1,16; *r* = 0.295; 95% CI [−0.19, 0.70]).

## Discussion

In both grazed and improved fallows, plant diversity displayed significant positive effects on ecosystem service multi-functionality. The strength of evidence for plant diversity effects on multi-functionality depended on fallow type. In grazed fallows there was stronger evidence for positive relationships of plant diversity with ecosystem multi-functionality, especially since environmental conditions could not provide a superior explanation. While significant effects of plant diversity on mean percentage excess were observed in improved fallows, environmental favorability appeared more influential.

In grazed fallows, both the proportion of ecosystem functions above half-maximum (“proportion above half-maximum”) and mean percentage excess above mean ecosystem function values (“mean percentage excess”) increased with functional diversity and species richness. The decline in the proportion above half-maximum with grazing intensity explained less of the variance than did plant diversity variables, and no such decline was observed for mean percentage excess. Positive effects of fallow plant diversity on ecosystem multi-functionality were stronger than negative effects of grazing, yet it was not unequivocally clear that diversity effects were independent of variation in grazing intensity. It remains possible that more intense grazing, perhaps in conjunction with variation in soil conditions, could have influenced plant diversity or altered diversity effects. Significant effects of functional diversity on multi-functionality in moderately grazed fallows indicated that within this group of fallows, effects of functional diversity on both the proportion above half-maximum and mean percentage excess were independent of grazing.

In improved fallows, there was mixed evidence for effects of plant diversity and environmental conditions on ecosystem multi-functionality. Improved fallows typically sustained at least two of the four ecosystem functions above half-maximum, irrespective of both diversity and soil carbon. In contrast, mean percentage excess increased with both diversity and soil carbon, with soil carbon out-performing diversity variables as a predictor. Positive effects of soil carbon on mean percentage excess indicated that multi-functionality may have been more easily attained under environmental conditions favorable for the production of biomass, development of soil structure and improvement of infiltration, and the deposition of base cations in litter and their retention in soils. Environmental favorability appeared more influential than plant diversity, and the potential of diversity to benefit multiple ecosystem functions in improved fallows was uncertain. In improved fallows soil carbon was positively though weakly associated with plant functional diversity (d.f. = 1,19; *r* = 0.202; 95% CI [−0.25, 0.58]) and species richness (d.f. = 1,19; *r* = 0.207; 95% CI [−0.25, 0.59]). Despite the weakness of these associations, greater diversity in more productive sites could have contributed to diversity effects on mean percentage excess.

### Jointness among ecosystem functions

Pair-wise jointness among ecosystem functions is a component of ecosystem multi-functionality as quantified here (Supplement S1). The infrequent jointness for wood and forage biomass in improved fallows reflected a trade-off attributable to large woody plants suppressing forage species growing beneath through competition for light and soil resources. In grazed fallows, jointness for wood and forage biomass was not particularly low, indicating that large woody plants did not compete strongly with forage species, failing to generate a wood-forage trade-off. The incidences of jointness for wood and forage biomass were consistent with correlations (negative in improved; positive in grazed) among the two functions (Figure 1). Although plant diversity may reduce the severity of trade-offs among ecosystem functions [13], linear co-variance generally could not explain jointness for most pairs of functions. The limited sample size posed a challenge to attributing jointness to biological mechanism.

The incidence of jointness was at times attributable to the probability distributions of ecosystem functions (Supplement S1). Returning to the example of jointness among soil base cations and steady infiltration rates, high jointness indicated that improved fallows were typically capable of sustaining the two elements of soil quality at moderate levels. As base cations did not correlate with soil carbon in improved fallows, there was no basis for attributing jointness among base cations and infiltration to linear influences of soil carbon. In grazed fallows, grazing may have reduced jointness among base cations and infiltration, for example through soil compaction due to livestock trampling (a likely cause of low infiltration in grazed fallows). Since grazing did not affect soil carbon, effects of soil carbon on infiltration may have been weaker under grazing. Base cations increased with soil carbon, as the two were highly correlated; as for carbon, grazing did not affect base cations. Despite no clear linear explanation, livestock trampling could have decoupled infiltration from soil carbon, thus reducing the degree to which higher values of base cations and infiltration co-occurred.

### Ecosystem multi-functionality: Grazed fallows

The results for grazed fallows indicate possible means by which plant diversity may have enhanced the capacity of fallow plant assemblages to sustain multiple ecosystem functions. Diversity may have increased multi-functionality through complementary benefits of species with different functional traits to different functions, and through effects of multi-functional species on more than one function. In addition, by elevating the importance of disturbance response traits or altering species interactions, grazing disturbance may have promoted plant diversity effects on multi-functionality.

While diversity may have positively affected ecosystem functioning through complementarity and sampling effects specific to individual ecosystem functions, multi-functionality should increase with representation of species that contribute to different ecosystem functions. Otherwise, individual functions could benefit from diversity, but diversity might not improve multi-functionality.

#### Complementarity among functions

Complementary effects of species on different ecosystem functions should exert additive effects on multi-functionality—that is, functions would exhibit trade-offs in accordance with the occurrence of species specialized in providing each function. If diversity did not influence multi-functionality through any other means (e.g., complementarity and/or sampling effects on individual functions, multi-functional species, disturbance-related effects), multi-functionality could be predicted from the values of individual functions alone. However, the presence of species providing complementary benefits to different functions may contribute, in part, to diversity effects on multi-functionality. Complementary effects of species on different functions may occur alongside complementarity effects specific to individual functions, and multi-functionality was expected to increase when species are complementary both within and among ecosystem functions.

In grazed fallows, wood originates from trees and shrubs, and forage primarily from herbaceous species. Different functional traits were associated with and may have benefitted these two components of fallow biomass. Wood biomass increased with green tissue content of lignins, and since wood declined with grazing intensity, the correlation between wood and tissue lignin suggests that less palatable woody plants were more successful in the presence of grazing. In contrast, forage biomass increased with green tissue N, and also declined with grazing intensity, suggesting that less palatable forage species prevailed under intense grazing. Thus, not only are wood and forage produced by different subsets of the assemblage, they appeared to increase with divergent functional traits, and therefore appear to benefit from plant species with complementary influences on wood versus forage production. As such, high levels of both wood and forage should co-occur where species have high content of either lignins or N. As calculated here, greater functional diversity would meet this condition, since high lignin values were uncommon, and herbaceous lignin content was particularly low.

While wood biomass increased with lignin content, polyphenol content was negatively though insignificantly associated with wood biomass. Since infiltration and soil carbon (which correlated with soil base cations) responded positively to vegetation with higher tissue content of recalcitrant C compounds—lignins+polyphenols in particular (J. Sircely, *unpublished data*)—the joint occurrence of high wood biomass with high infiltration and base cations may be more likely in plant assemblages including species with high content of either lignins or polyphenols. Again, such a combination of traits would coincide with higher functional diversity.

#### Multi-functional species

Species capable of effectively supporting more than one ecosystem function may have enhanced multi-functionality in grazed fallows. It is possible, if perhaps unlikely, that a few multi-functional species could provide sufficient levels of all ecosystem services demanded from a managed system, at least over finer temporal and spatial scales. However, such a system could be unrealistic in practice due to the probability of incomplete knowledge on species effects on each ecosystem service under particular abiotic and management regimes and combinations of species.

The increase in wood biomass with lignin content paralleled the positive influence of recalcitrant C compounds on soil conditions. In addition, some woody species have deep rooting systems, to 2.5 m in depth in the study area [39]. These species may prevent leaching of subsoil nutrients deeper into the soil profile [15], base cations included, and replenish soil nutrients through retrieval from subsoils and re-deposition in litter. Similarities in the traits linked with wood production and soil properties suggest possible overlap in the species benefitting these ecosystem functions.

Since both wood biomass and the proportion of functions above half-maximum declined with grazing intensity, the loss of potentially multi-functional woody species may have negatively influenced multi-functionality. The positive correlation between functional diversity and wood biomass indicated that plant diversity in grazed fallows increased with the representation of large woody plants, many of which can be considered multi-functional. The representation of multi-functional species in biotic communities may increase probabilistically with species richness, effectively generating a sampling effect on multiple functions. The presence of multi-functional species possessing unique combinations of functional traits should disproportionately increase functional diversity.

#### Disturbance

Echoing observations of diversity effects on individual ecosystem functions strengthening under disturbance [16], [17], more frequent and intense grazing may have intensified diversity effects on multi-functionality. Benefits of diversity to ecosystem resilience under disturbance and effects of grazing on species interactions may have strengthened the diversity-multi-functionality relationship.

The diversity in plant functional response traits—traits mediating responses to environmental change—influences ecosystem functioning by determining species composition [22]. Response traits providing tolerance to grazing and other disturbances tend to exhibit trade-offs at the species level with traits associated with avoidance of disturbance and re-colonization [29], [31]. Here, functional diversity includes response traits since tissue N, lignins, and polyphenols affect palatability and are associated with instantaneous growth rates. Greater response trait diversity may have enhanced the ability of fallow assemblages to maintain functioning under the consistent and at times severe disturbance from livestock grazing. That is, species with diverging response strategies may have improved the resilience of ecosystem functioning under grazing.

Species interactions significantly determine ecosystem effects of biodiversity [9]. Competition implies substantial niche overlap, indicating possible functional redundancy and limited complementarity, while positive interactions are a form of complementarity [8]. Lower dominance and competition should promote ecosystem effects of inferior competitors, including those supporting different ecosystem functions than dominants. Greater evenness and positive species interactions improve biomass production [40], likely stimulating soil functions as well. By reducing competition and increasing facilitation, grazing may have enhanced complementarity effects on individual ecosystem functions as well as complementary benefits of species to different functions.

The negative relationships of grazing intensity with tree and shrub biomass (d.f. = 1,16; *r* = −0.473; 95% CI [−0.77, −0.01]) and forage biomass (d.f. = 1,16; *r* = −0.486; 95% CI [−0.78, −0.02]) indicated that intense grazing reduced the biomass of the overstory as well as forage species, generating the positive association between wood and forage biomass (Figure 1). Grazing likely prevented overstory suppression of forage species by reducing woody recruitment and thinning the canopy, and may have reduced competition among fallow species in general.

Grazing may have also increased facilitation, since species less palatable to grazers often facilitate those more palatable [41] by impeding their detection and consumption. However, intense grazing weakens facilitation mediated by palatability [42], an effect that could have contributed to low multi-functionality in the most heavily grazed fallows. Abiotic stress can also shift species interactions from competition toward facilitation [43], [44], and poor site conditions may strengthen the diversity-function relationship [9]. Grazed fallows were sandier on average than improved, and their lower infiltration capacity (likely due to livestock trampling) may indicate restricted root access to water and nutrients, stressors that could reinforce positive interactions. Grazing may have enhanced the sensitivity of multi-functionality to diversity by reducing competition and inducing positive species interactions.

### Ecosystem multi-functionality: Improved fallows

In improved fallows, the fact that soil carbon best predicted mean percentage excess indicates that multi-functionality may be sensitive to the favorability of environmental conditions. Higher soil carbon is indicative of soil conditions that support biomass production as well as improvement in the structure and fertility of soils. Although the proportion of functions above half-maximum was unaffected by soil carbon, it is probably justifiable to conclude that environmental favorability enhanced multi-functionality, in part because mean percentage excess appeared to be a more robust indicator of multi-functionality (Supplement S1).

Beyond the evidence for environmental influences on multi-functionality, there are additional explanations for the weak and inconsistent diversity effects in improved fallows. A primary rationale for the use of improved fallows is their high productivity [15], [39]. Although explicitly quantifying productivity in these systems would require more detailed measures, productivity differences among fallow types are probable. Greater productivity in improved fallows would increase wood production, and greater inputs to soils should enhance soil and root function and improve soil fertility, structure, and infiltration capacity. Meanwhile, the apparent competitive suppression of forage species in the understory was attributable to the dominance of planted woody legumes. In dense, productive plant assemblages, the suppression of inferior competitors often reduces evenness, which can restrict complementarity effects [9], [40], [45]. Dominance may have weakened the diversity-multi-functionality relationship in improved fallows by limiting complementarity effects on individual functions, and by constraining functions supported by inferior competitors (e.g., forage biomass).

However, the species composition of improved fallows was not conducive to detecting diversity effects. The dominance of woody legumes and the commonness of *Tephrosia* fallows limited variation in plant diversity, functional traits, and the abundance of putative multi-functional species. Improved fallows varied little in community mean tissue contents of N, lignins, and polyphenols; limited variation in other functional traits is likely, e.g., nitrogen fixation capacity and root-to-shoot ratios. Significantly, the woody legumes in improved fallows are all somewhat multi-functional, and their consistent abundance may have obscured their potential to affect multi-functionality.

It is unknown whether species augmentation would improve the ability of improved fallows to sustain multiple ecosystem functions. Ndufa et al. (2009) conducted an experiment with improved fallow monocultures and two-species mixtures. While monocultures and mixtures produced similar amounts of biomass, mixed fallows out-performed monocultures on average in terms of N recycled to soils and post-fallow maize yields, although a monoculture performed best for both functions. Thus, while complementarity may enhance multiple ecosystem services in improved fallows, the potential may be limited and dependent on species composition.

### Synthesis: Management, biodiversity, and multi-functionality

The observed relationships among plant diversity, environmental conditions, and ecosystem service multi-functionality differed substantially between grazed and improved fallows. In grazed fallows, significant effects of plant diversity on multi-functionality indicators provided evidence in support of plant diversity enhancing the capacity of fallows to provide multiple ecosystem services. Diversity effects on multi-functionality were stronger and more consistent than negative effects of grazing intensity. In contrast, soil carbon appeared more influential than diversity in improved fallows, and evidence for diversity effects on multi-functionality was scant. The ecological factors influencing multi-functionality thus appeared to differ appreciably among fallow types, with plant diversity more influential in grazed fallows and environmental favorability more important in improved fallows.

Differences in management between grazed and improved fallows created contrasting ecological conditions that may explain much of the apparent divergence in ecological controls over ecosystem multi-functionality. The improved fallows here were ungrazed and experienced little disturbance, while grazed fallows were subjected to consistent disturbance from livestock grazing. The other major management difference was that improved fallows were intentionally planted with leguminous trees and large-statured shrubs, while the vegetation in grazed fallows reflected largely unguided regeneration. These management dissimilarities were likely responsible for generating the prevailing differences in vegetation structure and species composition among fallow types.

The results indicate that the management of smallholder fallows influences the relationship between plant diversity and ecosystem service multi-functionality. The productive, multi-functional species planted in improved fallows were highly dominant, generating competition and limiting variation in plant diversity, functional traits, and the abundance of multi-functional species. As a result, fallow management to create dense stands of woody legumes appeared to reduce or obscure the potential for plant diversity to affect multi-functionality. In contrast, grazing disturbance and unguided regeneration in grazed fallows led to variation in plant diversity, functional traits, and multi-functional species abundance, and reduced dominance and competition (and possibly increased facilitation). Effects of plant diversity on multi-functionality in grazed fallows therefore appear related to management of fallows with livestock grazing and without planting woody legumes. The results from grazed fallows are consistent with complementary benefits of different plant species to different ecosystem functions and multi-functional species being among the sources of positive influences of plant diversity on ecosystem service multi-functionality.

To improve understanding of the relationship between biodiversity and ecosystem service multi-functionality, research investigating sources of variation in the relationship is a sensible extension of the findings presented here. Disturbance and perturbations, environmental conditions, dominance, and species interactions—all recognized as significant in previous biodiversity-ecosystem function research—are promising avenues toward more fully elucidating the potential significance of plant diversity in sustaining the ability of agroecosystems to provide multiple ecosystem services, and the possible roles of biodiversity in maintaining the overall functioning of ecosystems managed and natural alike.

## Materials and Methods

### Study systems

The Millennium Villages Project (MVP) is an evidence-based approach to alleviating extreme rural poverty in sites throughout Sub-Saharan Africa, in which agricultural development plays a strategic role [46]. The data for this study were collected in 2008–2009 in the Sauri MVP site in Siaya District, western Kenya. The site has a humid tropical climate with two rainfall peaks and potential cropping seasons yearly, and ranges in elevation from 1,300 to 1,500 m. The clayey, well drained Oxisols and Ultisols are common soil orders in Sub-Saharan Africa. The landscape is a shifting matrix primarily composed of maize and other annual crops, with less cover of woodlots, grazing land, and fields in fallow. Land use cycles between annual crops and periods of fallow intended to regenerate soil fertility and to provide wood products, green manures, and livestock forage, among other goods. High population and cropping frequency and inadequate fertilization have led to soil degradation since the early 20^th^ century [44], and soils in the area remain depleted of N and C, but Sauri agroecosystems are undergoing long-term rehabilitation.

### Fallow types

‘Improved’ fallows of fast-growing leguminous trees and shrubs are promoted by MVP to improve soil fertility and produce fuelwood. Improved fallows in the area are primarily *Tephrosia candida* DC, and less commonly *Crotalaria paulina* Schrank, *Calliandra calothyrsus* Meissn. and other exotics, or the native *Sesbania sesban* (L.) Merr. Improved fallows in the present study were not grazed with livestock. Grazed fallows, often considered a type of ‘natural’ or ‘weed’ fallow, are less common, and contain a variety of mostly native species. Trees common in grazed fallows include the native *Sesbania* and *Markhamia lutea* (Benth.) K. Schum., and the exotic *Psidium guajava* L. Most grazed fallows combine production of wood and green manures with livestock grazing, although the most heavily grazed fallows produce little or no wood.

### Data collection

Fallow fields identified for sampling ranged in size from 0.02 to 0.25 ha, with a mean of approximately 0.1 ha. Data were collected from 18 grazed and 21 improved fallows using a down-scaled version of the Land Degradation Surveillance Framework (LDSF) protocol [47], modified for within-field sampling. The LDSF protocol characterizes vegetation structure (diameter at breast height (DBH) of trees, tree and shrub stem density, and vegetation cover), soil conditions, erosion, and FAO-standard land use/cover. Soils are sampled at depths of 1–20 cm (topsoil) and 20–50 cm (subsoil). The modified LDSF protocol was implemented in a 100 m^2^ plot located randomly in the interior of each fallow. Soil samples were collected and soil infiltration capacity measured in two 2 m^2^ subplots, one in the plot center, and one randomly located tangent to the plot edge. Biovolume (m^3^) of all vascular plant species was characterized by visually estimating cover and average height. Cover was scored as the mid-point of cover classes on a 5-point scale: 1–4, 4–15, 15–40, 40–65, and >65%. Grazing intensity was recorded by visually estimating cover for evidence of grazing—trampling, feces, bite marks, and hair—on the same 5-point scale, and fallows placed into groups of lightly (4–40%), moderately (40–65%), and heavily (>65%) grazed.

### Standing biomass—large woody stems

For all woody stems ≥2.5 cm DBH, aboveground standing biomass (t ha^−1^) was estimated by using DBH to calculate whole-tree biomass (*Y*) by the general allometric equation, *Y* = e^(−2.134+2.53 *ln*DBH)^ for humid regions [48]. For standing green (leaves and young green twigs) and woody biomass, aboveground biomass was multiplied by mean values from the literature of the proportion of aboveground biomass for tree species common in the study site (Table S2).

### Standing biomass—small woody stems and herbaceous

For all woody plants <2.5 cm DBH and all non-woody species biovolume (m^3^) was multiplied by a conversion factor to estimate aboveground biomass (Table S2). For standing green (leaves and young green twigs) and woody biomass, the same procedure was used as for aboveground biomass, using conversion factors that reflect aboveground green or woody biomass (Table S2).

### Soil properties

Soil organic carbon, soil base cations, and soil sand content were quantified using near infrared reflectance spectroscopy (NIRS), utilizing extensive soil libraries from the study site and the region to predict values of soil properties [49], [50]. Predictions were made using linear mixed effects models to predict soil carbon, base cations, and sand from the principal components (PCs) of first-derivative NIR spectral reflectance in the 700 to 2500 nm range. Top- and sub-soil were modeled as random effects. Cross-validation based on an independent sample set demonstrated model fit *r* ^2^ values of: 0.91 for soil organic carbon; 0.92 for Mg; 0.70 for Ca; 0.88 for K; and 0.92 for sand. Estimates of Mg, Ca, and K were summed to estimate base cations. Infiltration was measured with ring infiltrometers 20 cm in diameter over approximately 2.5 hours, or until steady state was reached. Soil infiltration capacity was quantified as steady infiltration rates (mm hour^−1^), the lower asymptote approached by infiltration rate over time. Each ∼2.5 hr measure was modeled as a random effect in a non-linear mixed effects model of the Horton equation [51], via asymptotic regression using the nlmer function in the lme4 package [52] in R software [53]. For each soil property the mean was taken for the 2 nested subplots.

### Plant traits

Samples of leaves and young green twigs (leaves only for herbaceous species) were collected from a subset of study plots, and NIRS was used to predict content of N, lignins and polyphenols for most common species. Predictions were made using partial least squares regression to model N, lignins, and total soluble polyphenols from PCs of first-derivative NIR spectral reflectance in the 1250 to 2500 nm range. Independent sample cross-validation yielded model fit *r* ^2^ values of: 0.98 for N; 0.73 for lignin; and 0.86 for polyphenols. Mean trait values were calculated by species. For species not sampled in study plots, mean trait values by species were obtained from the Organic Resource Database [54]. Community-weighted means (CWM) of trait values [55] were calculated from natural log-transformed species mean values for green tissue N, lignins, and polyphenols, weighted upon species standing green biomass. Abundance-weighted means among species of trees and large shrubs, and among forage species, were also calculated by weighting trait means on the green biomass of trees and large shrubs, and of forage species.

### Wood biomass

For the ecosystem service of wood production, the ecosystem service providers, or ESPs [56], were taken to be the species of trees and large shrubs that were sufficiently large in a plot to serve as fuelwood for cooking locally. Woody biomass of trees and large shrubs included individual stems ≥2.5 cm DBH, and all woody species with average height ≥0.7 m. With few exceptions, woody species of smaller stature provide insignificant quantities of useful wood.

### Forage biomass

For the ecosystem service of forage production, the ESPs were taken to be the forage species producing higher quality forage in terms of the green tissue crude protein (CP; i.e., the standard tissue N ×6.25) to lignin ratio [57], i.e., those useful for feeding livestock. While forage species included all herbaceous species and woody species <0.7 m average height, higher quality forage species had green tissue CP:lignin ≥0.8, which approximates the upper limit of the lowest quartile of CP:lignin values among species in the sample. All species in the sample known to be used for feeding livestock [58] had CP:lignin >0.8, while all species not known to be used as forage had CP:lignin <0.8. Forage species with low CP content relative to lignin are of little use as feed for livestock, as lignin impedes digestion and nutrient absorption. Forage biomass is indicative of the actual amount of standing biomass useful for feeding livestock.

### Species richness

Species richness was calculated as the number of species comprising ≥1% of standing green biomass in a fallow. The influence of species with low plot-scale abundance on ecosystem functioning is likely to be minor; these species disproportionately influence evenness and are likely to bias estimation of any influences of plant diversity on ecosystem functioning. Although species richness and evenness were negatively correlated, when species comprising <1% of green biomass were removed, the correlation was effectively eliminated.

### Functional diversity

Functional diversity (FD) was used as a proxy for niche complementarity among fallow plant species [59]. Because functional diversity reflects dissimilarity among the species in an assemblage, it can be considered a measure of complementarity [60], [61]. Calculation of functional diversity again included only species comprising ≥1% of standing green biomass in a fallow. Functional diversity was calculated as the summed branch length of trees generated by UPGMA average linkage clustering of Euclidean multivariate distances among species in terms of three functional traits: green tissue contents of N, lignins, and polyphenols. Each trait exhibited little correlation across fallow plant species. Trait values were natural log-transformed prior to calculations, and were re-scaled to a mean and standard deviation of 1. Functional diversity estimation was robust to trait selection; upon inclusion of maximum height, RSR, lignins+polyphenols, growth form, and woodiness, estimates of functional diversity remained tightly correlated with one another. As such, the conservative set of three traits was selected. In the study system, green tissue contents of N, lignins, and polyphenols are likely associated with rates of production and accumulation of biomass, biological nitrogen fixation, species effects on soil processes, competitive and facilitative species interactions, and responses to grazing. This conservative set of traits is therefore considered to contain significant information on complementarity among fallow plant species.

### Ecosystem service indicators

Ecosystem functions directly related to specific ecosystem services served as indicators of the focal ecosystem services, and each is relevant to the livelihoods of smallholder farmers in the study area. Because total standing green biomass and soil carbon were correlated to varying degrees with other ecosystem functions, collinearity among functions could have biased analysis of ecosystem multi-functionality. Instead, a conservative approach to ecosystem multi-functionality was adopted. The ecosystem functions selected for analysis (Table 1) were: standing woody biomass of trees and large shrubs (i.e., available wood), standing green biomass of forage species (i.e., available forage), soil content of base cations, and steady infiltration rates (i.e., two elements of soil formation).

In these agroecosystems, each ecosystem function reflects specific stocks or fluxes of energy, materials, or both. Wood biomass specifically indicates the stock of non-living aboveground biomass, and the potential flux of C, energy, and nutrients in wood to households for fuelwood, poles, etc. Forage biomass is the stock of biomass available for consumption by livestock, and reflects the potential flux of C, energy, and nutrients to livestock biomass and waste. Soil base cation content integrates the cycling, movements, and soil functions provided by Mg, Ca, and K, including cation retention and exchange, plant uptake, leaching and prevention thereof, and retrieval from the subsoil by deep-rooted plants. Soil infiltration capacity is indicative of robust soil structure, avoidance of runoff and soil erosion, and retention and availability of soil water to plants.

### Ecosystem multi-functionality

Fallow ecosystem service multi-functionality among the 4 selected ecosystem functions was quantified by means of 2 indicators: (1) the proportion of ecosystem functions above half-maximum (“proportion above half-maximum”), and (2) the mean percentage excess above mean function values (“mean percentage excess”). That is, “proportion above half-maximum” is the proportion of the 4 ecosystem functions that were higher than 50% of the respective maximum value of each function, and “mean percentage excess” is the mean of the percentage by which ecosystem functions exceeded their mean values. The maximum for each function in the grazed and improved fallow types was defined as the mean of the 3 highest values in each fallow type. One mean and one maximum was established for each function in each fallow type among the two sampling years, as forage biomass did not differ between 2008 and 2009 (not presented), and is the only function likely to vary across years with rainfall and other climatic conditions. The proportion of functions above 25 and 75% of maxima were also calculated. Ecosystem functions were transformed (Table 1) prior to calculation of multi-functionality indicators to minimize the influence of the probability distributions of ecosystem functions.

### Statistical analysis

All analyses were conducted with R software [53]. The response of ecosystem multi-functionality indicators to plant diversity variables and environmental variables was analyzed with univariate linear models for each plant diversity variable (functional diversity, species richness) and each environmental variable (soil carbon, grazing intensity), and with additive bivariate linear models comprising each unique combination of diversity variables and environmental variables (except for grazing intensity in improved fallows, which were ungrazed). Given the limited sample size and likely low statistical power, interaction terms were not specified. To support interpretation of the central analyses, the incidence of pair-wise ‘jointness’ among ecosystem functions was calculated. Jointness for a specific pair of ecosystem functions was defined as when the observed values of both functions were above 50% of respective maxima, or above mean values. Jointness among two ecosystem functions was interpreted as reflecting the observed ability of a fallow to simultaneously sustain the pair of functions at or above moderate levels of each.

## Supporting Information

Figure S1 **Variation among fallow types in ecosystem multi-functionality in terms of the proportion of ecosystem functions above 25, 50, and 75% of respective function maxima.**
(PDF)

Supplement S1 **Pair-wise jointness among ecosystem functions.**
(DOC)

Table S1 **Plant diversity and environmental variables serving as predictors of fallow ecosystem multi-functionality.**
(DOC)

Table S2 **Derivation of growth form-specific biovolume to biomass conversion factors.**
(DOC)
