# Supplementary material for: Biodiversity and Ecosystem Multi-Functionality: Observed Relationships in Smallholder Fallows in Western Kenya
Source: PLoS One. 2012 Nov 28;7(11):e50152. doi: 10.1371/journal.pone.0050152 (PMC3509158; doi:10.1371/journal.pone.0050152)
Supplement: Figure S1 — Variation among fallow types in ecosystem multi-functionality in terms of the proportion of ecosystem functions above 25, 50, and 75% of respective function maxima. (PDF) [file pone.0050152.s001.pdf]

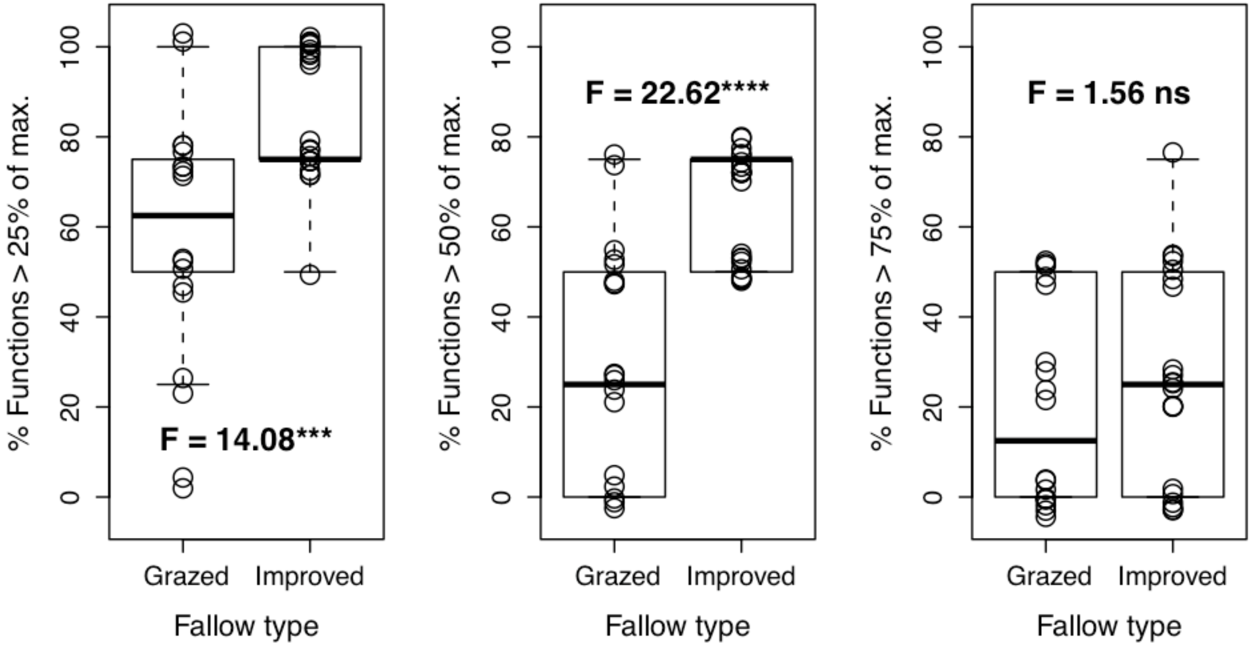

Figure S1 Variation among fallow types in ecosystem multi-functionality in terms of the proportion of ecosystem functions above 25, 50, and 75% of respective function maxima; points are jittered slightly for greater clarity. Statistics presented from one-way ANOVA with d.f. = 1, 37. * = $P < 0.05$, ** = $P < 0.01$, *** = $P < 0.001$, **** = $P < 0.0001$, ns = $P > 0.1$.
