# Supplementary material for: Biodiversity and Ecosystem Multi-Functionality: Observed Relationships in Smallholder Fallows in Western Kenya
Source: PLoS One. 2012 Nov 28;7(11):e50152. doi: 10.1371/journal.pone.0050152 (PMC3509158; doi:10.1371/journal.pone.0050152)
Supplement: Supplement S1 — Pair-wise jointness among ecosystem functions. (DOC) [file pone.0050152.s002.doc]

**Supplement S1.** Pair-wise jointness among ecosystem functions

Pair-wise jointness among ecosystem functions in the study fallows is a component of their ecosystem service multi-functionality, and facilitates interpretation of plant diversity and environmental influences on multi-functionality. Jointness for a specific pair of ecosystem functions was defined as when the observed values of both functions were above 50% of their respective maxima (for the proportion of ecosystem functions above half-maximum), or above mean values (for mean percentage excess above mean ecosystem function values). Jointness among two ecosystem functions was interpreted as reflecting the observed ability of a fallow to simultaneously sustain the pair of functions at or above moderate levels of each.

The term jointness draws upon discussions of the policy and management of multi-functional agricultural landscapes (Selman 2009, Turpin et al. 2010) capable of providing benefits beyond commodity production. Such landscapes are managed to provide for the conservation of biodiversity and the maintenance of soil formation, water quality, and other ecosystem services lacking markets. The original usage in microeconomics (Baumol 1981) refers to the ability of a firm to attain ‘economies of scope,’ the reduction in average cost per unit for a firm engaged in joint production of two or more products (‘economies of scale’ is more apt for a single product).

As defined here, the incidence of jointness is the result of co-variance among ecosystem functions and the statistical attributes of ecosystem functions, both of which potentially influence ecosystem multi-functionality. For instance, for two hypothetical ecosystem functions that are both random, normal, and uncorrelated, 25% of the sample is expected to support both functions jointly above 50% of their respective maxima, and above mean values. In a scatterplot of two such functions, the top-right quadrant of the figure would delimit the replicates achieving jointness. A strong correlation would affect jointness: positive co-variance would increase jointness, clustering replicates in the top-right and bottom-left quadrants, while negative co-variance would reduce jointness and cluster replicates at top-left and bottom-right. The shape of the probability distributions of ecosystem functions are another potential determinant of jointness. When two functions are both distributed asymmetrically and higher values of each co-occur, jointness will be higher, and vice versa. Thus, jointness arises from linear correlations among ecosystem functions suggestive of linear trade-offs (negative co-variance) or complementarities (positive co-variance) among ecosystem functions, as well as the probability distributions of ecosystem functions.

The low incidence of jointness for wood and forage biomass in improved fallows (Figure 5) reflected the negative correlation between the two functions (Figure 1). The marginally significant negative correlation between wood biomass and soil base cations in improved fallows may be related to their infrequent jointness with respect to mean values.

The incidence of jointness with respect to 50% of maximum differed substantially from jointness with respect to mean values. In particular, jointness above 50% of maximum was much more prevalent in improved than in grazed fallows, for nearly all pairs of ecosystem functions (Figure 5), while jointness above mean values did not exhibit any striking contrasts among fallow types. The probability distributions of ecosystem functions were generally skewed toward low values in grazed fallows (Figure S2), while in improved fallows, distributions were skewed to favor higher values (Figure S3).

Jointness above 50% of maximum was more strongly affected by the distributional characteristics of ecosystem functions than was jointness above mean values. In grazed fallows, skewed probability distributions of ecosystem functions toward lower values reduced the incidence of jointness at 50% of maximum. The opposite effect was observed in improved fallows, with skew toward higher values increasing jointness overall. For example, the incidence of jointness among soil base cations and steady infiltration rates illustrates the influences of the direction and degree of skewness in the distributions of ecosystem functions on the incidence of pair-wise jointness among ecosystem functions (see also in Results and Discussion). Base cations and infiltration were skewed toward low values in grazed fallows (Figure S2), and toward high values in improved fallows (Figure S3). Consequently, the incidence of jointness among base cations and infiltration was much lower in grazed than in improved fallows (Figure 5).

The absence of consistent differences in the incidence of jointness with respect to mean values among fallow types suggests that jointness above mean values was more robust to skewed distributions than was jointness at 50% of maximum. By extension, mean percentage excess above mean ecosystem function values may have served as a more robust indicator of ecosystem multi-functionality than the proportion of ecosystem functions above half-maximum.


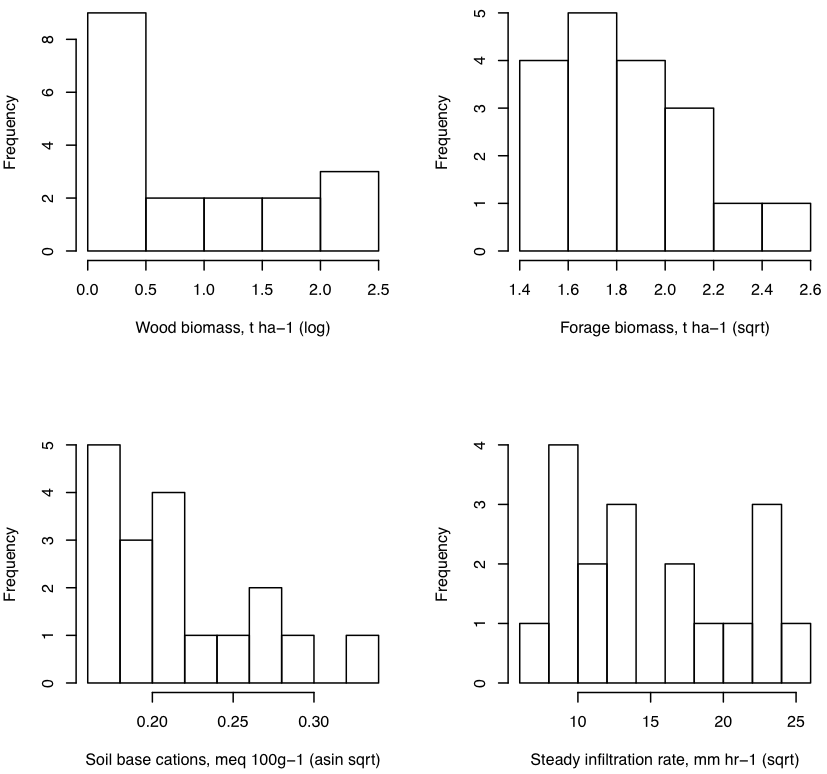


**Figure S2** Distributions of ecosystem functions (transformed) in grazed fallows.


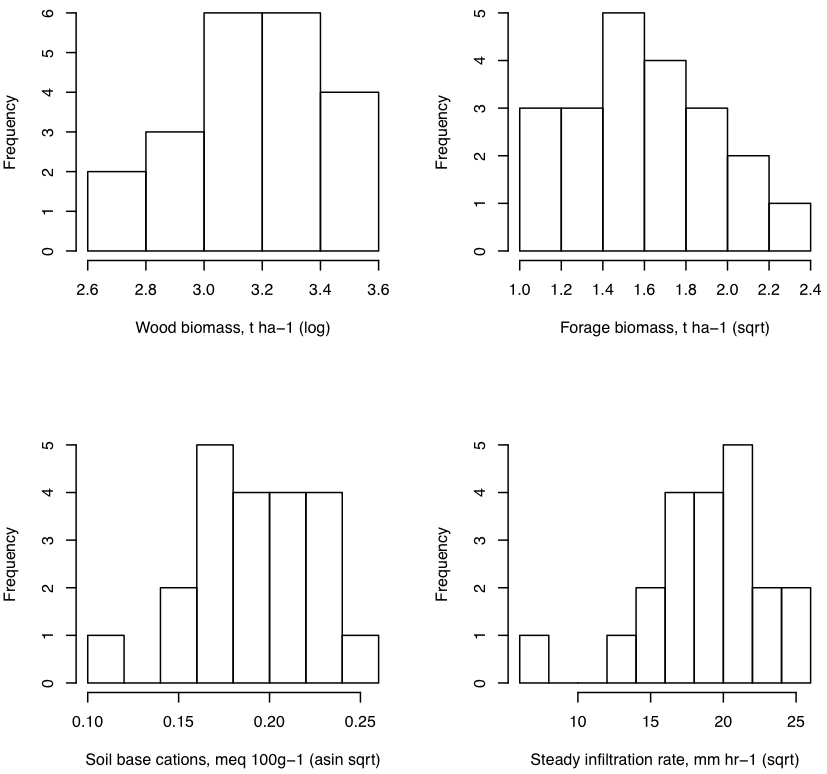


**Figure S3** Distributions of ecosystem functions (transformed) in improved fallows.

**References**

Baumol W, Panzar J, Willig R (1981) Contestable markets and the theory of market structure. New York: Harcourt, Brace and Jovanovich.

Selman P (2009) Planning for landscape multifunctionality. Sustainability: Science, Practice, & Policy 5: 45–52.

Turpin N, Stapleton L, Perret E, van der Heide CM, Garrod G, et al. (2010) Chapter 2: Assessment of Multifunctionality and Jointness of Production. In: Brouwer FM, van Ittersum M, editors. Environmental and Agricultural Modelling: Integrated Approaches for Policy Impact Assessment. Dordrecht, Netherlands: Springer. pp. 11–36.
