# Supplementary material for: Biodiversity and Ecosystem Multi-Functionality: Observed Relationships in Smallholder Fallows in Western Kenya
Source: PLoS One. 2012 Nov 28;7(11):e50152. doi: 10.1371/journal.pone.0050152 (PMC3509158; doi:10.1371/journal.pone.0050152)
Supplement: Table S1 — Plant diversity and environmental variables serving as predictors of fallow ecosystem multi-functionality. (DOC) [file pone.0050152.s003.doc]

**Table S1.** Plant diversity and environmental variables serving as predictors of fallow ecosystem multi-functionality.

| Variable type | Predictor | Units (Transformation) | Fallow type | n | Mean | SD | SEM |
| --- | --- | --- | --- | --- | --- | --- | --- |
| Plant diversity | Functional diversity | Unitless | Grazed | 18 | 2.4 | 1.2 | 0.3 |
|  |  | (none) | Improved | 21 | 1.7 | 1.1 | 0.2 |
| Plant diversity | Species richness | Counts | Grazed | 18 | 11.8 | 3.9 | 0.9 |
|  |  | (none) | Improved | 21 | 6.6 | 3.1 | 0.7 |
| Environmental | Soil organic carbon | % | Grazed | 18 | 1.5 | 0.3 | 0.1 |
|  |  | (arcsine square root) | Improved | 21 | 1.5 | 0.3 | 0.1 |
| Environmental | Grazing intensity | % area, approx. | Grazed | 18 | 60.3 | 22.5 | 5.3 |
|  |  | (arcsine square root) | Improved | na | na | na | na |
