# Supplementary material for: Biodiversity and Ecosystem Multi-Functionality: Observed Relationships in Smallholder Fallows in Western Kenya
Source: PLoS One. 2012 Nov 28;7(11):e50152. doi: 10.1371/journal.pone.0050152 (PMC3509158; doi:10.1371/journal.pone.0050152)
Supplement: Table S2 — Derivation of growth form-specific biovolume to biomass conversion factors. (DOC) [file pone.0050152.s004.doc]

**Table S2.** Derivation of growth form-specific biovolume (m^3^) to biomass (g) conversion factors (CFs) multiplied by species biovolume to estimate biomass.

| Fallow composition | Aboveground total biomass density, g m^-3^ | Aboveground green biomass density, g m^-3^ | Source |
| --- | --- | --- | --- |
| Mixed perennials, herbs dominant | 531 | 531 | Gathumbi et al. 2004 |
| Mixed perennials, herbs dominant | 545 | 545 | Ndufa et al. 2009 |
| Mixed perennials, herbs dominant | 472 | 472 | Mekonnen et al. 1997 |
| CFs for herbaceous species: | 516 | 516 |  |
| Mixed perennial herbs/sub-woody/woody | 332 | 207 | Ndufa et al. 2009 |
| Mixed perennial herbs/sub-woody/woody | 906 | 565 | Maroko et al. 1999 |
| Mixed perennial herbs/sub-woody/woody | 698 | 449 | Niang et al. 2002 |
| Mixed perennial herbs/sub-woody/woody | 898 | 577 | Niang et al. 2002 |
| Mixed perennial herbs/sub-woody/woody | 283 | 182 | Niang et al. 2002 |
| CFs for sub-woody species: | 623 | 396 |  |
| Mixed perennial herbs/sub-woody/woody | 623 | 396 | Ndufa et al. 2009; Maroko et al. 1999; Niang et al. 2002 |
| *Desmodium uncinatum* | 975 | 618 | Niang et al. 2002 |
| *Macroptilium atropurpureum* | 1042 | 660 | Ndufa et al. 2009 |
| *Macroptilium atropurpureum* | 417 | 264 | Ndufa et al. 2009 |
| *Macroptilium atropurpureum* | 375 | 238 | Gathumbi et al. 2004 |
| CFs for shrub species: | 708 | 449 |  |
| *Calliandra calothyrsus* | 949 | 464 | Ståhl et al. 2002 |
| *Crotalaria paulina* | 1000 | 476 | Ndufa et al. 2009 |
| *Crotalaria paulina* | 595 | 286 | Ndufa et al. 2009 |
| *Crotalaria paulina* | 1057 | 357 | Jama et al. 2008 |
| *Crotalaria paulina* | 557 | 191 | Gathumbi et al. 2004 |
| *Tephrosia candida* | 1184 | 343 | Jama et al. 2008 |
| *Tephrosia candida* | 678 | 196 | Niang et al. 2002 |
| *Sesbania sesban* | 1727 | 479 | Maroko et al. 1999 |
| *Sesbania sesban* | 1212 | 200 | Ndufa et al. 2009 |
| *Sesbania sesban* | 758 | 152 | Ndufa et al. 2009 |
| *Sesbania sesban* | 2406 | 448 | Niang et al. 2002 |
| *Sesbania sesban* | 2302 | 477 | Mekonnen et al. 1997 |
| CFs for large shrub and tree species: | 1288 | 353 |  |

**References**

Gathumbi SM, Cadisch G, Giller KE (2004) Improved fallows: effects of species interaction on growth and productivity in monoculture and mixed stands. Forest Ecology and Management 187: 267–280.

Jama BA, Mutegi JK, Njui AN (2008) Potential of improved fallows to increase household and regional fuelwood supply: evidence from western Kenya. Agroforestry Systems 73: 155–166.

Maroko JB, Buresh RJ, Smithson PC (1999) Soil phosphorus fractions in unfertilized fallow-maize systems on two tropical soils. Soil Science Society of America Journal 63: 320–326.

Mekonnen K, Buresh RJ, Jama B (1997) Root and inorganic nitrogen distributions in sesbania fallow, natural fallow and maize fields. Plant and Soil 188: 319–327.

Ndufa JK, Gathumbi SM, Kamiri HW, Giller KE, Cadisch G (2009) Do mixed-species legume fallows provide long-term maize yield benefit compared with monoculture legume fallows? Agronomy Journal 101: 1352–1362.

Niang AI, Amadalo BA, de Wolf J, Gathumbi SM (2002) Species screening for short-term planted fallows in the highlands of western Kenya. Agroforestry Systems 56: 145–154.

Ståhl L, Nyberg G, Högberg P, Buresh RJ (2002) Effects of planted tree fallows on soil nitrogen dynamics, above-ground and root biomass, N_2_-fixation and subsequent maize crop productivity in Kenya. Plant and Soil 243: 103–117.
